# Assessing User Experience and Satisfaction with a Mobile Application for Drug Dosage Calculation—A Pilot Study

**DOI:** 10.3390/dj14010020

**Published:** 2026-01-04

**Authors:** Rasa Mladenovic, Marko Milosavljevic, Zlatica Mirkovic, Kristina Mladenovic

**Affiliations:** 1 Department of Dentistry, Faculty of Medical Sciences, University of Kragujevac, 34000 Kragujevac, Serbia; drm.milosavljevic@yahoo.com; 2 Dental Medicine Clinic Dentokids, 34000 Kragujevac, Serbia; 3 Department of Internal Medicine, Faculty of Medicine, University of Pristina, 38220 Kosovska Mitrovica, Serbia; 4 Department of Physical Medicine and Rehabilitation, University Clinical Center of Kragujevac, 34000 Kragujevac, Serbia; kristinamladenovic1990@gmail.com; 5 Department of Physical Medicine and Rehabilitation, Faculty of Medical Sciences, University of Kragujevac, 34000 Kragujevac, Serbia

**Keywords:** pediatric dentistry, mobile application, drug dosage, patient safety, digital dentistry

## Abstract

**Background/Objectives**: Accurate drug dosage calculation in pediatric dentistry represents an essential component of everyday clinical practice. However, manual calculation methods, reliance on memory, and inconsistent pharmacological education often lead to uncertainty among practitioners. **Methods**: To support clinicians in this process, a mobile application—Dent.IN CALC—was developed as a rapid, evidence-based, and user-friendly tool. The app allows the input of age and weight to instantly generate recommended and maximum safe dosages of commonly prescribed antibiotics, analgesics, and local anesthetics. Additionally, it includes a list of corresponding pharmaceutical preparations available on the local market. A preliminary evaluation among sixty dentists revealed significant variability in dosage knowledge and confirmed the need for digital tools that facilitate accurate and efficient prescribing. **Results**: Most users rated the app as intuitive, time-saving, and highly beneficial for daily practice (mean satisfaction score 4.7 ± 0.4; 95% would recommend the app). **Conclusions**: The Dent.IN CALC app shows strong user acceptance and demonstrates how digital solutions can streamline workflow and support clinicians in routine pediatric pharmacological decision-making.

## 1. Introduction

Accurate dosage calculation in pediatric dentistry remains a daily challenge for clinicians, primarily due to considerable variability in body weight, metabolic rate, and developmental physiology among children [1,2,3]. Pediatric patients differ fundamentally from adults in terms of pharmacokinetics and pharmacodynamics—processes that influence drug absorption, distribution, metabolism, and excretion. Consequently, medications that are safe and effective in adults cannot simply be prescribed in reduced quantities for children; instead, each dose must be calculated individually based on age, weight, and clinical condition.

Even minor miscalculations in pediatric dosing can lead to significant clinical consequences. Underdosing may result in therapeutic failure, inadequate infection control, or prolonged recovery, whereas overdosing carries the risk of systemic toxicity, allergic reactions, or drug-induced complications. These concerns are particularly relevant in pediatric dentistry, where commonly used medications—such as local anesthetics, analgesics, and antibiotics—have relatively narrow therapeutic safety margins.

Recent studies highlight that a substantial proportion of dental practitioners experience difficulties in determining appropriate drug dosages for children [1,2]. This challenge is closely related to the limited inclusion of pediatric pharmacology in undergraduate and postgraduate dental curricula. Many programs still place greater emphasis on adult pharmacotherapy, providing insufficient training in child-specific dose adjustment, contraindications, and pharmacovigilance principles. This educational gap may contribute to avoidable prescribing errors, particularly among general practitioners who occasionally treat children but do not specialize in pediatric care.

Furthermore, the lack of easy access to up-to-date dosage guidelines during daily clinical work remains a critical barrier. Clinicians often face time pressure and must make rapid decisions in time-constrained environments, where manual calculation methods or consulting printed pharmacological tables can be impractical. These manual approaches—especially when performed under stress—carry a high risk of error, particularly when practitioners rely on outdated notes, approximate estimations, or verbal information provided by pharmaceutical representatives.

Reliance on memory or non-standardized conversion formulas has also been identified as one of the most common causes of preventable medication errors in pediatric healthcare. A landmark study by Kaushal and colleagues reported that nearly one-third of pediatric medication errors occur during the dosage calculation phase, most frequently due to conversion or unit errors, underscoring the need for systematic support tools in clinical practice [4]. Similar trends have been observed in dental settings, where inaccurate estimation of maximum anesthetic doses or antibiotic regimens poses a direct threat to patient safety.

In recent years, the integration of digital technologies into healthcare has emerged as a promising solution for minimizing medication errors. Mobile health (mHealth) applications and digital decision-support systems provide clinicians with rapid, standardized, and evidence-based recommendations at the point of care, improving calculation accuracy while also reinforcing pharmacological knowledge [5]. Reflecting this trend, this short communication describes the development and preliminary evaluation of Dent.IN CALC, a mobile application designed to assist pediatric dental clinicians in accurately calculating drug dosages during clinical practice.

## 2. Materials and Methods

### 2.1. Application Development

The Dent.IN CALC mobile application (available at: https://bit.ly/DentINCALC, accessed on 10 November 2025.) was conceptualized and developed by a pediatric dentist (first author Rasa Mladenovic) to address the everyday clinical need for accurate, rapid, and standardized drug dosage calculation in children. The app was developed for Android operating systems using the Android Studio Integrated Development Environment (IDE) (Google, Mountain View, CA, USA). The frontend interface was coded in HTML5 and XML, while the core logic and functionality were implemented using Java programming language (version 17).

**Figure 1:**
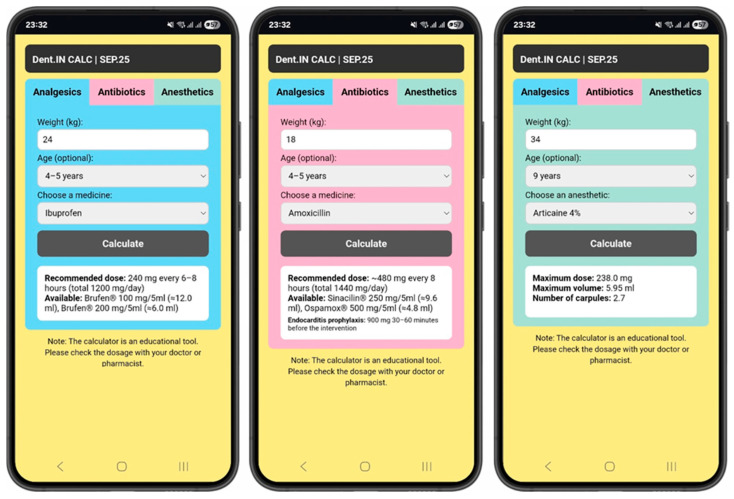
Screenshots of Dent.IN CALC application.

The user interface (UI) was designed following Google’s Material Design guidelines, emphasizing clarity, accessibility, and fast navigation. The interface uses clearly labeled data input fields for a child’s age (years) and body weight (kilograms), along with dynamically generated dosage outputs (Figure 1). The backend algorithm integrates pharmacological reference data and calculation formulas derived from official pediatric dosage guidelines and the American Academy of Pediatric Dentistry (AAPD) Reference Manual (Latest Revision) [6,7].

Data processing within the app is performed entirely locally on the device, ensuring user privacy and eliminating the need for an internet connection during clinical use. The app’s architecture allows for future integration of additional drug databases and multilingual support through modular updates. Calculations were performed by pediatric dentists and pharmacists for all available inputs and validated by comparison with standard AAPD formulas and patient information leaflet.

### 2.2. Core Functionalities

- Patient-specific input: clinicians enter the child’s weight (kg) and age (years).
- Instant calculation: the algorithm provides both recommended and maximum safe doses for key pharmacological groups—antibiotics, analgesics, and local anesthetics.
- Market relevance: the app includes pharmaceutical formulations available in the local market, helping clinicians quickly match calculated doses with available commercial preparations.
- Safety features: where applicable, the app displays alerts when the maximum safe dose is approached or exceeded, reinforcing safe prescribing behavior.
- The visual interface uses large, clearly labeled buttons and dosage fields for rapid use, even during clinical procedures. The app’s offline functionality is particularly beneficial in dental offices or regions with limited internet connectivity, ensuring reliability in all practice settings.

### 2.3. Study Design and Evaluation Procedure

A cross-sectional pilot study was conducted in September 2025 to evaluate the clinical utility, usability, and perceived educational value of the Dent.IN CALC application. The evaluation involved 60 dental practitioners, including general dentists, pediatric dental specialists, and practitioners from other dental disciplines. Participants were recruited via professional mailing lists and social media groups of dental associations. The study was conducted on a non-probability convenience sample of 60 participants, all from Serbia or the broader former Yugoslav region. This geographic concentration was primarily due to the language of the Dent.IN CALC application and all study materials, which were available exclusively in Serbian. Consequently, participation was restricted to practitioners fluent in Serbian, limiting the potential for broader international recruitment. Participants were recruited voluntarily using a convenience sampling method. Included were licensed dentists actively engaged in clinical practice, while those not currently working in a clinical setting were excluded. This approach ensured that the study population consisted solely of practicing dental clinicians capable of evaluating the Dent.IN CALC application.

As this was a pilot study aimed at assessing usability, clinical utility, and perceived educational value, 60 participants were considered sufficient to provide preliminary insights and identify potential areas for improvement in the application. The response rate calculation was based on the number of participants who completed the questionnaire out of those who voluntarily agreed to participate, as invitations were disseminated via professional mailing lists and social media groups where the total number of recipients could not be precisely determined. This study was conducted using an anonymous questionnaire without collecting any personal, identifiable, or sensitive data, and therefore, in accordance with institutional guidelines, did not require ethical approval. Each participant voluntarily downloaded and used the application over a two-week period before completing an anonymous online questionnaire created in Google Forms. The questionnaire was adapted specifically for this study, based on previously published similar instruments [8]. The questionnaire assessed the following:

- Demographic and professional characteristics (specialization, years of experience, workplace setting);
- Self-assessed knowledge of pediatric pharmacology and dosage calculation;
- Satisfaction with the app’s usability, accuracy, and speed; and
- Perceived clinical and educational value.

Responses were recorded using a five-point Likert scale (1 = strongly disagree, 5 = strongly agree) for satisfaction items.

### 2.4. Statistical Analysis

Data obtained from the Google Forms questionnaire were analyzed using IBM SPSS Statistics v27.0. Descriptive statistics (frequencies, percentages, means, standard deviations, and 95% confidence intervals) were applied to summarize responses. Differences between groups by specialization and years of experience were assessed using the Chi-square test, Mann–Whitney U test, and Kruskal–Wallis H test, with a significance level set at *p* < 0.05. Effect sizes were calculated for significant differences (r for Mann–Whitney U). The internal consistency of the Likert-scale items was evaluated using Cronbach’s alpha (α = 0.89), confirming high reliability of the questionnaire.

## 3. Results

A total of 60 dental practitioners participated in the pilot evaluation. Of these, 38 (63.3%) were general dentists, 14 (23.3%) pediatric specialists, and 8 (13.3%) belonged to other specialties. Regarding experience, 14 (23.3%) had less than 5 years in practice, 19 (31.7%) had 5–10 years, 16 (26.7%) had 10–20 years, and 11 (18.3%) had more than 20 years. Most participants (70%) worked in private practice (Table 1).

### 3.1. Educational Gap

The majority of participants (75%) indicated that their undergraduate education provided insufficient instruction on pediatric drug dosage calculation. Only 12 respondents (20%) reported feeling confident in performing manual calculations. Younger practitioners (<5 years of experience) demonstrated slightly higher confidence (30%) compared to more experienced colleagues (>5 years, 18%) (Table 2). A Chi-square test comparing confidence by years of experience showed no statistically significant difference (*p* = 0.24), suggesting that confidence levels were not significantly associated with experience in this sample.

### 3.2. Information Sources

Regarding sources of pharmacological information, slightly more than one quarter of respondents (28%) reported consulting official dosing manuals, while over one third (35%) relied primarily on online resources. Pharmaceutical brochures were used by 22% of participants, and 15% depended mainly on peer advice. These findings highlight the lack of standardized pharmacological references in daily dental practice (Table 3).

### 3.3. User Experience

Nearly all participants (93%) described the Dent.IN CALC app as very easy or extremely easy to use, and 90% appreciated the clarity of its interface. A large proportion (88%) reported reduced anxiety related to dosage errors. The mean satisfaction score was 4.7 ± 0.4 (95% CI: 4.6–4.8) on a five-point Likert scale. Pediatric dental specialists rated the app’s clinical relevance slightly higher (mean 4.8, 95% CI: 4.7–4.9) than general practitioners (mean 4.6, 95% CI: 4.5–4.7). A Mann–Whitney U test confirmed this difference was statistically significant (*p* = 0.03), with a medium effect size (r = 0.31) (Table 4).

### 3.4. Correlation with Experience

Dentists with 5–10 years of practice showed the highest appreciation of the app’s educational potential (92%), while those with >20 years valued its practicality and time efficiency most. A Kruskal–Wallis test across experience groups for perceived educational value was statistically significant (*p* = 0.048), indicating that early-career dentists rated the app as more educationally useful.

### 3.5. Benefits

Participants highlighted faster workflow, higher confidence, and standardized dosing. Suggestions included expanding the database (68%), adding dosing modules for medically compromised patients (45%), multilingual support (38%), and EHR integration (22%). Ninety-five percent would recommend the app to colleagues, and 87% would use it routinely. (Table 5).

### 3.6. Clinical Significance

The implementation of the Dent.IN CALC application in pediatric dentistry has multiple clinical benefits:

- Enhanced patient safety: by minimizing the likelihood of calculation errors and overdosing.
- Improved efficiency: dosage results are available instantly, optimizing workflow and chairside decision-making.
- Educational support: young clinicians gain confidence and understanding of pediatric pharmacology through practical application.
- Standardization: the app promotes consistency in prescription practices across clinicians and institutions.

## 4. Discussion

This pilot study demonstrates the importance of precise pediatric drug dosing in dentistry and highlights the potential role of digital tools such as Dent.IN CALC. Drug dosing in children requires particular precision due to the narrow therapeutic range of many medications and the physiological variability associated with growth and development. Even minor deviations in calculation can lead to clinically significant underdosing or overdosing, with consequences such as inadequate infection control, treatment failure, or toxic reactions [1,2,3].

Several studies have shown that pediatric medication errors represent one of the most frequent categories of preventable adverse events in clinical medicine, particularly in primary care and dental settings [9,10]. In pediatric dentistry, these risks are further intensified by the fact that dentists often prescribe to children only occasionally, limiting the experiential reinforcement of correct dosing. Moreover, differences in available formulations, units (mg/mL, mg/kg), and variations in product concentrations can easily lead to confusion during manual calculations [10]. In this study, 80% of participants reported low confidence in manual dose calculation, underscoring the persistence of these risks in daily dental practice. Consistent with the findings of Kaushal et al. [4], our results confirm that dosage miscalculations remain common due to unit conversion errors and insufficient pharmacological training.

The significance of precise dosage determination extends beyond pharmacological accuracy; it is also a matter of professional responsibility and patient safety. The World Health Organization (WHO) has repeatedly emphasized that medication safety is a critical component of global public health strategies, with pediatric patients representing one of the most vulnerable populations [11]. This is particularly relevant for dental practitioners, who may need to prescribe antibiotics and analgesics under time constraints and without immediate access to pharmacological databases.

Digitalization in healthcare offers a clear and practical solution to this challenge. The integration of clinical decision-support systems (CDSS) and mobile applications has been shown to reduce medication errors and improve prescribing accuracy across various medical specialties [12,13]. Mobile tools like Dent.IN CALC align with these global initiatives by providing evidence-based, user-friendly, and instantly accessible dosage recommendations that substantially decrease the risk of human error. In our study, 93% of participants rated the app as easy to use, and 88% reported reduced anxiety when prescribing medications for children.

Beyond immediate safety benefits, such digital tools have strong educational value. They function as dynamic learning platforms, reinforcing correct pharmacological reasoning through repeated clinical use. Previous research has demonstrated that mobile medical applications enhance clinicians’ self-efficacy and retention of drug-related knowledge, particularly among young or early-career professionals [14]. In our study, participants with fewer than five years of experience rated the app’s educational value highest (92%), indicating its potential as a learning-support tool for early-career dentists and residents.

Additionally, the application contributes to workflow optimization and reduction in cognitive load in demanding clinical environments. In pediatric dental settings—where clinicians must simultaneously manage patient behavior, communication, and procedural tasks—having instant access to accurate dosage information can significantly improve efficiency. The incorporation of such applications into routine practice may also reduce dependence on pharmaceutical representatives and promote the use of independent, evidence-based prescribing principles.

Finally, the broader implications of this innovation extend beyond pediatric dentistry. The methodology behind Dent.IN CALC can be adapted for general medicine, pharmacy, and nursing, contributing to the global movement toward safer, standardized, technology-assisted prescribing. The future of dental pharmacology will likely rely on the integration of clinical expertise, digital platforms, and artificial intelligence. As AI-based algorithms evolve, they may enable even greater personalization of dosage recommendations by incorporating patient-specific variables such as renal function, pharmacogenomic profiles, or comorbidity-adjusted pharmacokinetics [15,16,17,18]. Although these capabilities extend beyond the current scope of the app, future AI integration could allow for individualized, precision-based dosing in dentistry.

This pilot study has several limitations, including a small sample size, reliance on self-reported data, and the absence of objective performance testing. Future studies should involve larger, multi-center evaluations and real-time clinical validation.

Future development will focus on expanding the pharmacological database, refining dosage recommendations for special-needs and medically compromised patients, and creating an iOS-compatible and multilingual version of the app. Overall, the Dent.IN CALC application demonstrates measurable potential to improve prescribing safety, clinician confidence, and workflow efficiency in pediatric dental practice.

## 5. Conclusions

The Dent.IN CALC mobile application provides an evidence-based and user-friendly solution to one of pediatric dentistry’s key challenges—accurate, individualized drug dosage calculation. Its early adoption among clinicians demonstrated high user satisfaction and notable perceived educational benefits. These findings suggest that Dent.IN CALC can support clinical practice and contribute to the professional development of pediatric dental practitioners. Future research should evaluate the app’s effectiveness in larger, diverse clinical populations and explore integration with emerging technologies such as AI and pharmacogenomics to further enhance personalized pediatric care.

## Figures and Tables

**Table 1:** Demographic characteristics of the participants (*n* = 60).

Variable	Category	*n* (%)
**Specialization**	General dentists	38 (63.3)
	Pediatric dental specialists	14 (23.3)
	Other specialties	8 (13.3)
**Years of experience**	<5 years	14 (23.3)
	5–10 years	19 (31.7)
	10–20 years	16 (26.7)
	>20 years	11 (18.3)
**Practice setting**	Private practice	42 (70)
	Public sector	18 (30)

**Table 2:** Educational background and confidence in dosage calculation.

Parameter	Response	*n* (%)
**Undergraduate education on pediatric dosing**	Insufficient	45 (75)
	Sufficient	15 (25)
**Confidence in manual dosage calculation**	Confident	12 (20)
	Not confident	48 (80)
**Confidence by experience group**	<5 years	(30%)
	>5 years	(18%)

**Table 3:** Sources of pharmacological information used in practice.

Source of Information	% of Respondents (*n* = 60)
**Official dosing manuals**	28
**Online resources**	35
**Pharmaceutical brochures**	22
**Peer advice**	15

**Table 4:** User experience and perceived app performance.

Source of Information	Mean ± SD/%	Notes
**Ease of use (“very easy” or “extremely easy”)**	92%	-
**Clarity of interface**	90%	-
**Reduced anxiety regarding dosing errors**	88%	-
**Mean satisfaction score (Likert 1–5)**	4.7 ± 0.4	95% CI: 4.6–4.8
**Clinical relevance rating**	Pediatric specialists: 4.8; General dentists: 4.6	Mann–Whitney *p* = 0.03, r = 0.31

**Table 5:** Perceived benefits and suggested improvements of the app.

Aspect	Response Rate (%)	Description
**Faster workflow and standardized dosing**	100	Universally noted
**Increased professional confidence**	92	Common benefit
**Expand drug database**	68	Suggested improvement
**Add modules for medically compromised**	45	Suggested improvement
**Multilingual support**	38	Suggested improvement
**Integration with EHR systems**	22	Suggested improvement
**Would recommend the app to colleagues**	95	-
**Would use the app routinely**	87	-

## Data Availability

The original contributions presented in this study are included in the article. Further inquiries can be directed to the corresponding author.

## References

[1] Goel D., Goel G.K., Chaudhary S., Jain D. (2020). Antibiotic prescriptions in pediatric dentistry: A review. J. Fam. Med. Prim. Care.

[2] Carrasco-Labra A., Polk D.E., Urquhart O., Aghaloo T., Claytor J.W., Dhar V., Dionne R.A., Espinoza L., Gordon S.M., Hersh E.V. (2023). Evidence-based clinical practice guideline for the pharmacologic management of acute dental pain in children: A report from the American Dental Association Science and Research Institute, the University of Pittsburgh School of Dental Medicine, and the Center for Integrative Global Oral Health at the University of Pennsylvania. J. Am. Dent. Assoc..

[3] Mladenovic R. (2022). A simple calculation of the maximum dose of the local anesthetic in pediatric dentistry with nomogram. Vojnosanit. Pregl..

[4] Kaushal R., Bates D.W., Landrigan C., McKenna K.J., Clapp M.D., Federico F., Goldmann D.A. (2001). Medication errors and adverse drug events in pediatric inpatients. JAMA.

[5] Chau R.C.W., Cheng A.C.C., Mao K., Thu K.M., Ling Z., Tew I.M., Chang T.H., Tan H.J., McGrath C., Lo W.L. (2025). External Validation of an AI mHealth Tool for Gingivitis Detection among Older Adults at Daycare Centers: A Pilot Study. Int. Dent. J..

[6] American Academy of Pediatric Dentistry (2024). Use of local anesthesia for pediatric dental patients. The Reference Manual of Pediatric Dentistry.

[7] American Academy of Pediatric Dentistry (2022). The Reference Manual of Pediatric Dentistry, 2022–2023: Definitions, Oral Health Policies, Recommendations, Endorsements, Resources.

[8] Mladenovic R., Milosavljevic M., Stanisic D., Vasovic M. (2023). Importance of artificial intelligence in the analysis of children’s CBCT imaging by dental students. J. Dent. Educ..

[9] Rishoej R.M., Almarsdottir A.B., Christensen H.R., Hallas J. (2021). Medication errors in pediatric inpatients: A systematic review. Eur. J. Pediatr..

[10] Ghaleb M.A., Barber N., Franklin B.D., Yeung V.W., Khaki Z.F., Wong I.C.K. (2006). Systematic review of medication errors in pediatric patients. Ann. Pharmacother..

[11] World Health Organization (2022). Medication Safety in High-Risk Situations.

[12] Jia P., Zhang L., Chen J., Zhao P., Zhang M. (2016). The Effects of Clinical Decision Support Systems on Medication Safety: An Overview. PLoS One.

[13] Khairat S., Marc D., Crosby W., Al Sanousi A. (2018). Reasons for physicians not adopting clinical decision support systems: Critical analysis. JMIR Med. Inform..

[14] Masters K., Ellaway R.H., Topps D. (2020). Mobile technologies in medical education: AMEE Guide No. 105. Med. Teach..

[15] Shortliffe E.H., Sepúlveda M.J. (2018). Clinical decision support in the era of artificial intelligence. JAMA.

[16] Joshi S., Sheth S. (2025). Artificial Intelligence (AI) in Pharmaceutical Formulation and Dosage Calculations. Pharmaceutics.

[17] El Naqa I., Murphy M.J. (2021). Artificial Intelligence in Medicine and Radiation Oncology.

[18] Siontis G.C.M., Sweda R., Noseworthy P.A., Friedman P.A., Siontis K.C., Patel C.J. (2021). Development and validation pathways of artificial intelligence tools evaluated in randomised clinical trials. BMJ Health Care Inform..

